# On computational models of theory of mind and the imitative reinforcement learning in spiking neural networks

**DOI:** 10.1038/s41598-024-52299-7

**Published:** 2024-01-23

**Authors:** Ashena Gorgan Mohammadi, Mohammad Ganjtabesh

**Affiliations:** Department of Computer Science, School of Mathematics, Statistics, and Computer Science, College of Science, University of Tehran, Tehran, Iran

**Keywords:** Learning algorithms, Computational neuroscience, Network models

## Abstract

Theory of Mind is referred to the ability of inferring other’s mental states, and it plays a crucial role in social cognition and learning. Biological evidences indicate that complex circuits are involved in this ability, including the mirror neuron system. The mirror neuron system influences imitation abilities and action understanding, leading to learn through observing others. To simulate this imitative learning behavior, a Theory-of-Mind-based Imitative Reinforcement Learning (ToM-based ImRL) framework is proposed. Employing the bio-inspired spiking neural networks and the mechanisms of the mirror neuron system, ToM-based ImRL is a bio-inspired computational model which enables an agent to effectively learn how to act in an interactive environment through observing an expert, inferring its goals, and imitating its behaviors. The aim of this paper is to review some computational attempts in modeling ToM and to explain the proposed ToM-based ImRL framework which is tested in the environment of River Raid game from Atari 2600 series.

## Introduction

Theory of Mind (ToM) is the ability of inferring and/or attributing mental states (including knowledge, beliefs, goals, and intentions) to others and oneself. This ability allows individuals to make assumptions about the goals and intentions of others, facilitating the prediction of their subsequent actions^1,3–6^. Therefore, ToM plays an essential role in our social cognition and our interaction with the environment. It also affects our knowledge and beliefs about the world and how we learn through our lifespan from people around us^4,7,8^.

Despite its significance, the underlying mechanisms of ToM are not yet fully understood. Numerous studies have implicated brain regions such as Temporal Parietal Junction (TPJ), precuneus, Superior Temporal Sulcus (STS), Inferior Parietal Lobule (IPL), Inferior Frontal Gyrus (IFG), Premotor Cortex (PMC), and medial Prefrontal Cortex (mPFC) in activities related to ToM^3,7,9–11^. Among these regions, the STS is associated with the encoded visual perception, while the others are more linked to belief reasoning, self-other distinction, visual access, and motor responses.

More importantly, the Mirror Neuron System (MNS) is a crucial element enabling the imitation of behaviors within ToM^7^. First discovered in PMC of the Macaque monkeys in 1978, mirror neurons are activated not only when the animal performs an action, but also when he observes others doing a similar or identical actions^1,12^. Later and with the advances in brain imaging techniques, similar pattern was observed in human brain^1,3^. Also, the neuromodulation and lesion studies confirm the contribution of the MNS in imitation and goal-directed actions^13^. The MNS and the resultant imitative behavior in social decision making and Autism spectrum disorders (a disorder traditionally intertwined with ToM, but there is a running debate^2^) is an evidence of its contribution to ToM^17^. The MNS was believed to be the core circuitry of ToM; however, recent studies have argued that although MNS is an essential low-level infrastructure of ToM, higher levels of ToM involves more complex circuits, including mechanisms of imagination and memory^14,15^. Nevertheless, the MNS is a crucial element in imitation and action understanding as it originates from low-level ToM^3,7^, hence a good starting point to study ToM.

Given the computational challenges associated with designing a trainable model of the complete ToM circuit, the focus of this paper is on modeling the MNS. Therefore, a ToM-based Imitative Reinforcement Learning (ImRL) framework, inspired by the MNS, has been proposed in this paper, enabling an agent to imitate and learn the appropriate actions by observing other agents. The framework is suggested in the context of bio-inspired spiking neural networks (SNN) for two main reasons. First, SNNs take both spatial and temporal information into account and hence make them good candidates to model time-sensitive behaviors of ToM and MNS. Second, the associative sequence learning cognitive model of imitation can be easily demonstrated by the Spike-Timing Dependent Plasticity (STDP)^7^. This time-dependent extension of the Hebbian learning rule has previously been employed to model the emergence of mirror neurons^10^. Drawing inspiration from this work, the MNS is encapsulated in a two-layer SNN in the proposed model, comprising the STS and PMC. The PMC predominantly represents the mirror mechanism and contains one neuron per each possible action. The ToM-based agent is presumed to possess basic knowledge about the environment, referred as prior knowledge in this paper. This prior knowledge provides a foundation for Reinforcement Learning (RL) to be applied, assisting the imitative behavior of the MNS by offering cues about appropriate or inappropriate behaviors in the environment.

Over the last two decades, the advancements in AI and ToM findings have brought together scientists from diverse disciplines to propose computational models of ToM. In this paper, we review some of these attempts and argue that despite extensive research on the Mirror Neuron System (MNS) and its relation to ToM, none of the proposed models has been primarily centered around these findings. We then hypothesize that the ToM-based ImRL framework inspired by the work of Keysers and Gazzola (2014)^10^ can be used in practice to speed up training of RL tasks. To test this hypothesis, the proposed framework is trained and evaluated in the environment of Atari 2600 River Raid game^18^. It is shown that given an expert River Raid player, the behavior of ToM-based agent utilizing the input-output SNN of ToM-based ImRL framework resembles the expert’s behavior, as if the ToM-based agent has learned by figuring out the expert’s intentions and goal behind each action. We then illustrate that the performance gap between the ToM-based agent and the RL-based agents with identical SNN structure and weight initialization is significant. This indicates that the ToM-based agent can learn faster and more effectively by observing an expert. Finally, we compare ToM-based ImRL and the so-called imitation learning, followed by the arguments about the shortcomings of the proposed framework and the further study prospects.

## Background

Most of the computational models of ToM are evaluated by the extent to which they can pass the false belief tasks. In the false belief tasks, a situation is provided in which the examinee has full access to the available information in the environment, but there is a third person who has partial knowledge due to an unseen modification in the environment. To pass the test, the examinee should be able to inhibit their beliefs and decide based on the third person’s beliefs about the environment^4,19^. While most computational studies design a protocol to validate their model with this psychological ToM evaluation method, not all studies employ it to assess their models. The models designed to operate in more general purpose and real-world-like scenarios offer other metrics of evaluation.

Having that said, the first computational model of ToM is based on the first-order logic which explains the behavior under the false belief task^20^. Later, Bayesian models were proposed for planning and decision making under uncertainty, resulting in the famous model of Bayesian ToM^21–23^. The Bayesian ToM was the best computational model of ToM for years and its extensions were also proposed to describe different aspects of ToM^24,25^. This trend vanished in 2018, when Google’s DeepMind proposed ToM-net, the first artificial neural network model of ToM based on meta-learning^26^. ToM-net assesses the false belief task in a grid world, in which the states are partially-observable for other agents, but fully-observable by the learning agent. ToM-net would then be able to distinguish the false belief of other agents.

At the same year, ToM-Dyna-Q was proposed which combines the Dyna-Q architecture with ToM simulation in the Tic-Toc-Toe game^27^. This model is not developed to solve the false belief task, but to allow the learning agent to plan his actions based on simulating sequences of action-state-reward. In another study, the consequence engine was suggested as a computational model of ToM^5^. All the above mentioned studies pioneered a wider range of research to find out ToM potentials in AI and its computational benefits.

In 2019 and 2020, more research were performed to show how ToM can contribute to AI and what problems it can solve in this fast-paced developing world of AI^5,6,19^. For instance, deep learning methods have been paraphrased in ToM and mental simulation terms, to explain imagination and learning capacities^28^. Also, the Inverse Reinforcement Learning (IRL) was proposed as a way to formalize ToM in learning agents, represent beliefs and desires, and emulate human action understanding^29^. IRL algorithms try to predict the optimal policy in an environment where there is no explicit reward function. In this context, inferring other’s mental state from their observable behavior is analogous to predicting a reward function and the corresponding policy based on observations.

Despite the success of these models in justifying different behavioral and psychological aspects of ToM, none of them is biologically-plausible, in the sense that they are not based on neuronal mechanisms found in the biological brain. Brain ToM, a bio-plausible framework of ToM, fills this gap by helping humanoid robots solve the false belief task^11^. Nearly all the involved brain regions in ToM (as explained earlier) are modeled in Brain ToM framework. A variant of spiking neural networks, termed as VPSNN^30^, has been utilized to model precuneus and Anterior Cingulate Cortex (ACC), simulating the learning of visual access and object permanence in a robot. Except the STS, in which the template matching algorithm and Fast R-CNN are utilized to process information, the other brain regions are modeled with simple linear functions of their inputs. Consequently, a robot having Brain ToM would be able to pass the designed variant of the false belief task by only learning how to process the visual information and make a distinction between the self and the others^11^.

In a more recent work, ToM-SNN model was proposed to reduce safety risks posed by false beliefs of other agents in the environment^31^. ToM-SNN is a multi-area coordination model composed of different spiking modules, each of which corresponds to a specific brain region in ToM circuitry. These modules include a perspective taking, policy inference, action prediction, state evaluation, and decision, representing IFG and TPJ, vmPFC, dlPFC, dACC, and modulation of PFC with Dopamine from VTA/SNc, respectively. In this design, synaptic connections in the policy inference, action prediction, and state evaluation modules are plastic; the first two modules are trained by R-STDP learning rule and the latter is trained using STDP. This model is assessed in a grid world by a metric of safety risk, which is correlated by the number of collisions with other agents in the environment and the number of times the agent is hit by other agents^31^.

Even though Brain ToM and ToM-SNN are bio-plausible frameworks, they do not account for imitative learning and the MNS mechanisms explicitly. The trend of computational ToM for imitative learning was highlighted in 2021 by two main studies. In the 38th International Conference on Machine Learning, a ToM-based model was proposed for the purpose of few-shot language coordination. The study employs a DAgger-like algorithm of imitation learning which optimizes the prediction of linguistic actions conditioned on observations and instructions^32^. In another study, the CogToM framework, utilizing the instance-based learning theory of decisions, was tested in a gridworld, where the model learns from observation of goal-directed agent’s behavior^33^. While these studies offer algorithms of imitation learning based on ToM, they are not inspired by the biological neural mechanisms of MNS. This is where the proposed ToM-based ImRL framework comes into play to fill the gap.

## Proposed framework

The main objective of ToM-based ImRL is to increase the learning capacity of agents in an interactive environment by simulating low-level ToM and the MNS mechanisms, and empowering them with an imitative learning methodology. ToM-based ImRL is substantially based on two assumptions:

1. In an interactive environment and in the presence of an expert taking actions in it, an intelligent agent can learn how to interact with the environment in different circumstances by observing the expert, inferring the goals and intentions behind the expert’s actions, and imitating his behavior in similar situations.
2. An intelligent agent is able to distinguish rewarding and unrewarding/punishing behaviors given a description of the environment, where reinforcement learning plays an important role to enable reward-motivated learning.

A ToM-based agent can be placed in an interactive environment and observe the expert interacting with the environment. The ToM-based agent observes the action taken by the expert at current state and the consequence of this action in the environment, evaluates this action by its self-assumed reward value (assumption 2), and learn how to interact with the environment (assumption 1). The mirroring of the expert’s action is achievable by forcing the corresponding neuron to emit a spike (just as the mirror neurons do). So, whenever the expert’s action is rewarding based on ToM-based agent’s knowledge, the corresponding connections in the ToM-based agent’s neural network will be potentiated to increase the probability of choosing that action further when it interacts with the environment itself. Otherwise, the corresponding action will be depressed, to decrease the probability of selecting this action later. Meanwhile, the ToM-based agent would decide on an action solely based on its own knowledge. The corresponding connections to this action also should be potentiated in this case (or depressed in the former case) to give it a chance to be selected, in the hope that this would be a better action choice than what the expert had chosen in a similar situation.

Employing an SNN would result to have both spatial and temporal resolutions in the procedures of learning and acting. Having encoded the sensory input into spike trains, the temporal difference of pre- and post-synaptic neural populations could be effective in the learning procedure, due to the STDP learning rule (see Methods). On the other hand, the second assumption can be met by reward-modulated STDP (R-STDP) in the ToM-based agent. This design provides a good demonstration of ToM and the MNS in the sense of associative sequence learning cognitive model of imitation and enables the framework to form a representation of the observed actions in the mirror neurons located in PMC, a similar mechanism to low-level ToM^7,10^. The activation time of the output layer (or the post-synaptic population), hence, plays a crucial role in the training procedure. This timing should be selected regarding the information about the environment, which is compatible with the ToM-based agent’s prior knowledge.

## Experimental setup

For the purpose of this study, a learning agent is located in an environment of Atari 2600 River Raid game (see Methods for details). To explain the mentioned process in more details, the network architecture and training procedure designed for this chosen environment are provided in following subsections after a short description of the tools and materials employed in the simulations (also, a simpler experiment on the Cart and Pole environment can be found in Supplementary Information).

### Tools and materials

For the simulations in this study, the River Raid Atari game environment provided by OpenAI Gym is utilized^18^. For the expert, the provided model in Tensorpack is employed^34^, which is trained using Asynchronous Advantage Actor-Critic (A3C) algorithm^35^. This expert is trained on a batch of 128 recent states in each iteration, providing a rich history in the course of learning, and it can achieve the average score of 14185 over 100 independent runs. It has full access to a game frame, including the bar in the lowest part (see Fig. 5).

As mentioned earlier, ToM-based ImRL framework is an input-output SNN (see “Methods” for more information about SNNs). In this setting, the input indicates the processed visual information in STS, and the output represents the mirror mechanism in PMC, simplified as mirror neurons. The BindsNET framework is utilized to implement the desired SNN, since it provides wrappers on OpenAI Gym environments and facilitates the experiment design^36^.

### Network architecture

The simple input-output architecture in this study is inspired by the work of Keysers and Gazzola (2014)^10^. To demonstrate the full structure of the proposed model for a ToM-based agent located in the environment of Atari 2600 River Raid game (see Methods), one must first understand the road map of encoding the visual perception of this environment into interpretable spatiotemporal neural representations. Each frame of the game is divided into three groups of components, namely the obstacles in the scene (referred to as obstacles), the green margins at the sides of the river (referred to as side margins), and the oriented green margins in front of the plane (referred to as oriented margins). Each group will later be interpreted as a neural population, in which a component in the group and its location in the frame are represented by a neuron. A spike of a neuron will then be interpreted as the presence of that specific component in that particular location.

To encode this information into spike events, the first step involves extracting the components in each group, along with the plane and its shot, for each frame using convolutional template matching. It is noteworthy that the plane itself is not explicitly encoded; instead, all other components are encoded relative to it. To this end, after determining the coordinate of each component, its horizontal distance from the plane is calculated. Given that the vertical position of the plane remains fixed, the vertical coordinates of the components are directly utilized. To ensure precision and emphasize the significance of components closer to the plane while also reducing the number of utilized neurons, a linear transformation is applied to the distances greater than or equal to 20 pixels from the plane (summarizing every 4 pixels into one). In more mathematical terms, let $x_c$ and $y_c$ be the coordinate of component *c* in a game frame, and let $x_{p}$ and $y_{p}$ be the coordinate of the plane. Each component *c* will be represented by two distance values, namely $d_x(c,p)$ and $d_y(c,p)$, as follows:

1a $$\begin{aligned} d_x(c,p)&= {\left\{ \begin{array}{ll} |x_c - x_{p}| &{}\quad \text {if}\quad |x_c - x_{p}| < 20,\\ 20+\lfloor \frac{|x_c - x_{p}|}{4}\rfloor &{}\quad \text {otherwise}; \end{array}\right. } \end{aligned}$$

1b $$\begin{aligned} d_y(c,p)&= {\left\{ \begin{array}{ll} y_c &{}\quad \text {if}\quad |y_c - y_{p}| < 20,\\ 20+\lfloor \frac{y_c}{4}\rfloor &{}\quad \text {otherwise}. \end{array}\right. } \end{aligned}$$

Without loss of generality, it is assumed that the green margins are visible whenever they lie within specific horizontal distances from the plane. This assumption is made to control the number of input spikes and reduce redundant information derived from prior knowledge about the environment, directly impacting the population size. The designated distances for side and oriented margins are set to 8 and 20 pixels, respectively. These values have been determined experimentally and appear to strike the optimal balance between training time and performance. Side margin data is only determined for a total of 25 pixels above and below the location of the plane to avoid redundant information.

Additionally, a receptive field with radius of 10 pixels has been employed for the shot, ensuring the precision of shooting an obstacle. In other words, the distances of obstacles within a 10 pixel radius above the shot, shown by $d_x(c,s)$ and $d_y(c,s)$, are calculated by:

2a $$\begin{aligned} d_x(c,s)&= {\left\{ \begin{array}{ll} x_c - x_{s} &{}\quad \text {if}\quad |x_c - x_{s}| < 10,\\ \text {undefined} &{}\quad \text {otherwise}; \end{array}\right. } \end{aligned}$$

2b $$\begin{aligned} d_y(c,s)&= {\left\{ \begin{array}{ll} y_c - y_{s} &{}\quad \text {if}\quad 0< y_c - y_{s} < 10,\\ \text {undefined} &{}\quad \text {otherwise}; \end{array}\right. } \end{aligned}$$

where $x_{s}$ and $y_{s}$ are the coordinates of the shot. Also note that the plane and its shot are vertically aligned and $x_{s} = x_{p}$; so the two can be used interchangeably.

As a result, for each group of components, we respectively have a three-dimensional tensor of shape (*n*, *h*, *d*), where *n*, *h*, and *d* respectively denote the number of features (7 for obstacles, 4 for side margins, 3 for oriented margins, and 1 for shot distances), the height of the frame, and the maximum horizontal distance from the plane/shot. All together, there would be four independent input populations: one for the obstacles of shape (7, 101, 175), one for the side margins of shape (4, 25, 17), one for the oriented margins of shape (3, 101, 41), and one for the distance of obstacles from the shot of shape (1, 101, 21). The lowest bar in the game frame is not considered in ToM-based agent’s visual access to reduce the complexity of the network’s input (see Fig. 5).

**Figure 1:**
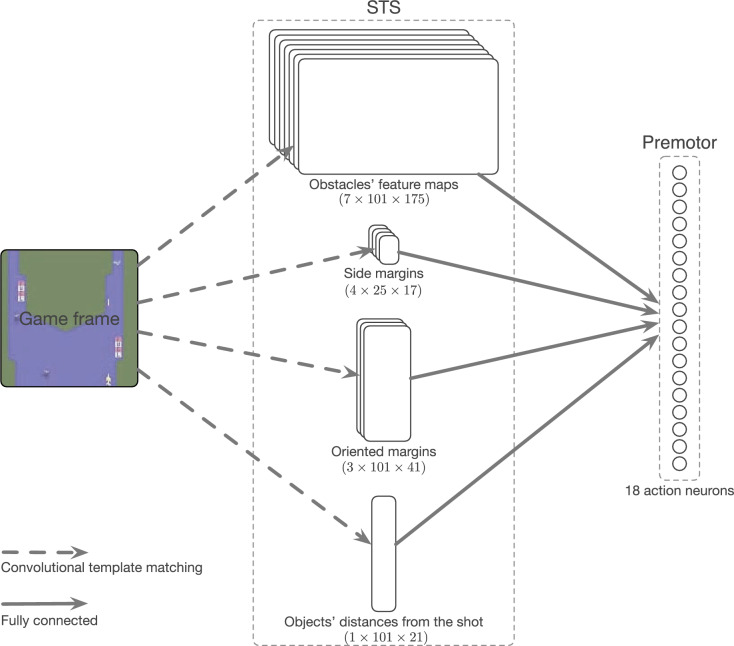
The schematic representation of the network architecture. Input layer (STS) consists of different neural populations, each corresponding to a group of components in the game. The neurons in this layer have no dynamics and only pass the spike trains to the output action layer, composed of LIF neurons. The information from each game frame is extracted via convolutional template matching. These spatial information of components is then encoded into explicit spikes over the time. In other words, the neuron corresponding to the presence of a component in a region of the game frame will spike at a time correlated with its vertical distance from the plane; making it spike earlier if it is within a shorter distance from the plane.

To define the structure of the output, all the possible actions in the environment should be considered. So, the output population of our network is composed of 18 LIF neurons, each for one of the possible actions (see Figs. 1 and 3b). Note that the optimal approach would involve considering a neuronal population for each action to more accurately represent the MNS. However, adopting such an approach would increase the number of connections and, consequently, the required computational resources. To mitigate these additional complexities, a voltage-based decision-making criterion is employed. This criterion, to some extent, is equivalent to deciding based on the firing rate of neuronal populations. The decision criterion is defined as the presence of a neuron with a voltage higher than $\alpha \cdot \bar{v}(t)$, where $\bar{v}(t)$ is the average of neural membrane potentials for the whole population at time step *t*, and $\alpha$ is a trainable parameter of the adaptive decision criterion in ToM-based ImRL framework. If the condition is met, the action neuron with the maximum membrane potential would be the winner and emits a spike. To this end, a high spike threshold is set for the output neurons to prevent them from firing and being reset.

The fully-connected scheme is considered between the input and the output populations. Given the assumptions of the ToM-based ImRL framework, as outlined in the Proposed Framework section, the synaptic plasticity applied to the connections in this network is evolved according to the R-STDP learning rule (except for the connections from the side margins to the output, where the weights are immutable). To fully leverage available information about the environment (see “Methods”), certain prior knowledge is embedded in the connections of the ToM-based agent and its reward policy. Specifically, if the frame is frozen, one of the lives is lost which is an unfavorable incident. Conversely, both surviving and shooting the obstacles are regarded as rewarding behaviors, with the latter being more pleasant and deserving of a higher reward. The reward policy is defined accordingly as below:

3 $$\begin{aligned} r(t) = {\left\{ \begin{array}{ll} -1 \quad &{}\text {if the frame is frozen},\\ 1 \quad &{}\text {if the shot hits an obstacle},\\ 0.1 \quad &{}\text {otherwise (surviving)}, \end{array}\right. } \end{aligned}$$

where reward of $-1$ indicates a punishing signal.

Since each population corresponds to a group of components, toward which a specific behavior should be taken, different ranges are proposed for their synaptic weights to demonstrate the suitable excitatory and inhibitory effects. These ranges are considered as $[-1, 1]$, $[-2, 1]$, and [0, 0.5] respectively for the obstacles, oriented margins, and distance from the shot. To regard the collision deterrence, synaptic weights are initialized relatively. As mentioned earlier, we fix the synaptic weights for the side margins. Consequently, the actions for moving toward the margins are highly inhibited when the margin is visible. For the oriented margins at closer vertical distances, moving toward them is inhibited while the opposite movements are excited. A similar inhibitory weight pattern is utilized for the obstacles to prevent the plane to approach them. For the missiles, moving in the direction of their movement in close distances is also inhibited to minimize the risk of collision. These inhibitions are graded within the 20 pixels distances in both horizontal and vertical directions; meaning the connections of neurons representing closer distances to the plane are more negative than the ones of far distances, while the synaptic weights of neurons of distances more than 20 pixels are set to zero.

### From observation to learning

The preceding discussion has elucidated the translation of input frames into neural populations and spike events. However, an essential aspect that remains unaddressed is the temporal coding of the input, a facet that significantly impacts both the performance of the ToM-based agent within the environment and, more prominently, the training process. Essentially, if the output neurons are activated within a short temporal distance after the input neurons, it would indicate that the respective components within the given distances were more influential on that decision. As a result, the corresponding synaptic weights are potentiated proportional to the timing of the pre- and post-synaptic spikes. Notably, components situated in closer proximity to the plane are more likely to fall within this influential temporal window. Conversely, the components that are farther away have less impact on the decision and their corresponding connections are depressed. Consequently, the timing of spikes from input neurons becomes inherently tied to their vertical distance from the plane.

The training protocol of ToM-based ImRL framework is composed of two phases, namely inferring and learning. During the inferring phase, the agent makes its own decision based on the observation but performs no action. This is analogous to mental simulation in humans. Let $A_m$ denote the action decided by the ToM-based agent and $t_d$ denote the decision time. Subsequently, in the learning phase, the actual imitative learning process unfolds.

**Figure 2:**
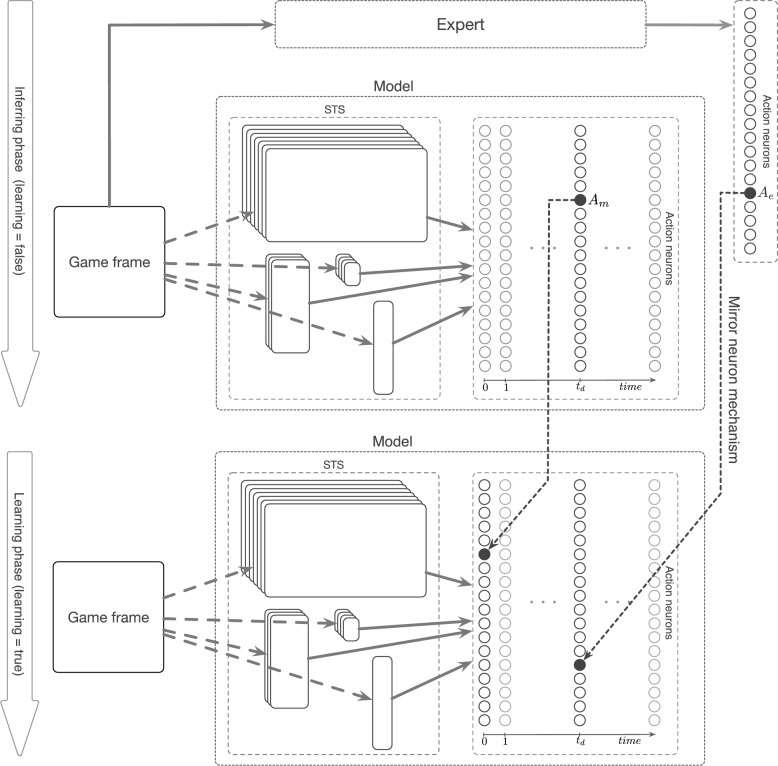
An overview of the ToM-based ImRL framework. The first pass demonstrates the actions, chosen by the expert and the ToM-based agent for the current frame of the game; while the second pass indicates the actual learning process, depressing contradicting self action and potentiating the mirrored expert’s action.

At the decision time of ToM-based agent ($t_d$), the mirror neuron corresponding to $A_e$, would be forced to spike. Forcing a spike is achieved by explicitly setting the corresponding element in the spike vector to 1. As a result, the synapses are strengthened accordingly (see Fig. 2).

There is also another mechanism involved during an imitative learning process in MNS. While observing another agent doing an action, the neurons corresponding to observer’s own action that are in contradiction with the observed action are inhibited. To achieve a similar behavior to this inhibition of self-action in the model, the neuron corresponding to ToM-based agent’s action ($A_m$, where $A_m\ne A_e$) is forced to spike before any other event in the learning phase, resulting in synaptic depression due to STDP.

Note that all these processes would be inverted in case of the expert’s failure (reward of $-1$). Hence, the connections to the neuron corresponding to $A_m$ will be potentiated to favor for it still being a possible good action, while the connections to $A_e$ are mainly depressed. More explicitly, the synaptic weight change at each time step would be defined by:

$$\begin{aligned} \frac{dw(t)}{dt} = r(t).c(t), \end{aligned}$$

where *r*(*t*) is computed by Eq. (3) and the dynamics of *c* (i.e. the synaptic tag) is defined in Eq. (4).

As mentioned above, the closer components to the plane have more impact on ToM-based agent’s decision, and the weight changes are dependent on the ToM-based agent’s decision time and the polarity of the reward. This information can guide the ToM-based agent through learning the points of attentions. So, for the ToM-based agent to learn the points of attention, an adaptive parameter $\alpha$ is deployed in the course of learning. If a contradicting action is chosen by the ToM-based agent, $\alpha$ is increased, forcing the agent to decide later in time in future. On the other hand, $\alpha$ is decreased if it can not make a decision itself according to the decision condition, enabling it to infer the expert’s goals and attention points sooner. More precisely, $\alpha$ will be modified as follows:

$$\begin{aligned} \frac{d\alpha (t)}{dt} = {\left\{ \begin{array}{ll} \mathrm {+1e}{-4}&{}\quad \text {if}\; A_m\ne A_e,\\ 0 &{}\quad \text {if}\; A_m=A_e,\\ \mathrm {-1e}{-4}&{}\quad \text {if no decision}. \end{array}\right. } \end{aligned}$$

Consequently, the ToM-based agent gradually learns what information was more involved in the expert’s action in each game frame. It infers the expert’s goals and motives resulting in the observed behavior, and tries to imitate this behavior by doing similar actions in analogous circumstances.

## Results

### Performance of the ToM-based agent

For the purpose of the experiments in this study, the input spikes are presented in a duration of 21 ms. As discussed in the previous section, each input neuron corresponds to a component and its position in the environment relative to the plane. This neuron would then spike earlier if it also denoted a position close to the plane. More precisely, the spike time of each neuron in the time range of [0, 20] ms is computed by:

$$\begin{aligned} t_{x,y}(c) = \min \{\lfloor \frac{y_c}{5}\rfloor , 18\} + 1 \end{aligned}$$

where *c* is one of the environment’s components, $x\in \{d_x(c,p),d_x(c,s)\}$ and $y \in \{d_y(c,p),d_y(c,s)\}$, all achievable by Eqs. (1) and (2). So, $t_{xy}(c)$ indicates the spike time of the neuron corresponding to component *c* at position (*x*, *y*) relative to the plane/shot. The values are shifted by 1, so that the 0 and 20 milliseconds will not be occupied by input spikes. As a result, $A_m$ will spike at the 0 millisecond when $A_m\ne A_e$ and $A_e$ can spike at the 20th millisecond if the ToM-based agent cannot decide its action in $t_d \le 19$. With respect to this duration, the time window of the STDP is set to 20 ms and the time constant of the eligibility trace is assigned to 400 ms, aligned with the synaptic tags of the last 20 frames.

The ToM-based agent observes the expert for 350 episodes, while the learning is enabled. We then let the ToM-based agent play the game and interact with the environment based on what it learned. With this setup, the ToM-based agent could score as high as 8860 and an average of 5597 over 100 independent runs. The score improvement from around the 100th episode is not significant and the enhancements mostly contribute to a more smooth behavior. This implies that only as few as 350 episodes suffice for the ToM-based agent to learn the expert’s strategies.

By observing the behavior of the expert, one can realize that the expert tries to shoot all the obstacles in the frame and does not make any distinction between the fuel tank and others. Additionally, it seems that the expert hits a margin intentionally, when low in fuel, has lives left, and has achieved a great amount of scores in the last stage. This strategy looks to be greedy, but it is helpful in guiding the expert through achieving higher scores. The goal and intentions of the expert are interpretable with respect to the mentioned facts and one would expect the ToM-based ImRL to infer these mental states and behave accordingly. To see if this is the case, we recorded the behavior of the ToM-based agent.

By observing the ToM-based agent’s behavior in the environment, one can notice that it tries to shoot any object it faces. This is inline with the expert’s behavior, which is shooting as much obstacles as possible to achiev higher scores; as if the ToM-based agent has realized that the motivation behind the expert’s behavior. Moreover, due to its prior knowledge about how to avoid collisions, it knows how to overcome a situation where it is too close to a component. Even though the expert does not usually move close to the obstacles and tries to shoot them from distance, there is a chance that the ToM-based agent finds itself in such circumstances. As a result, the ToM-based agent learns when to value its own assumptions, and when to inhibit its own knowledge and act like the expert.

**Figure 3:**
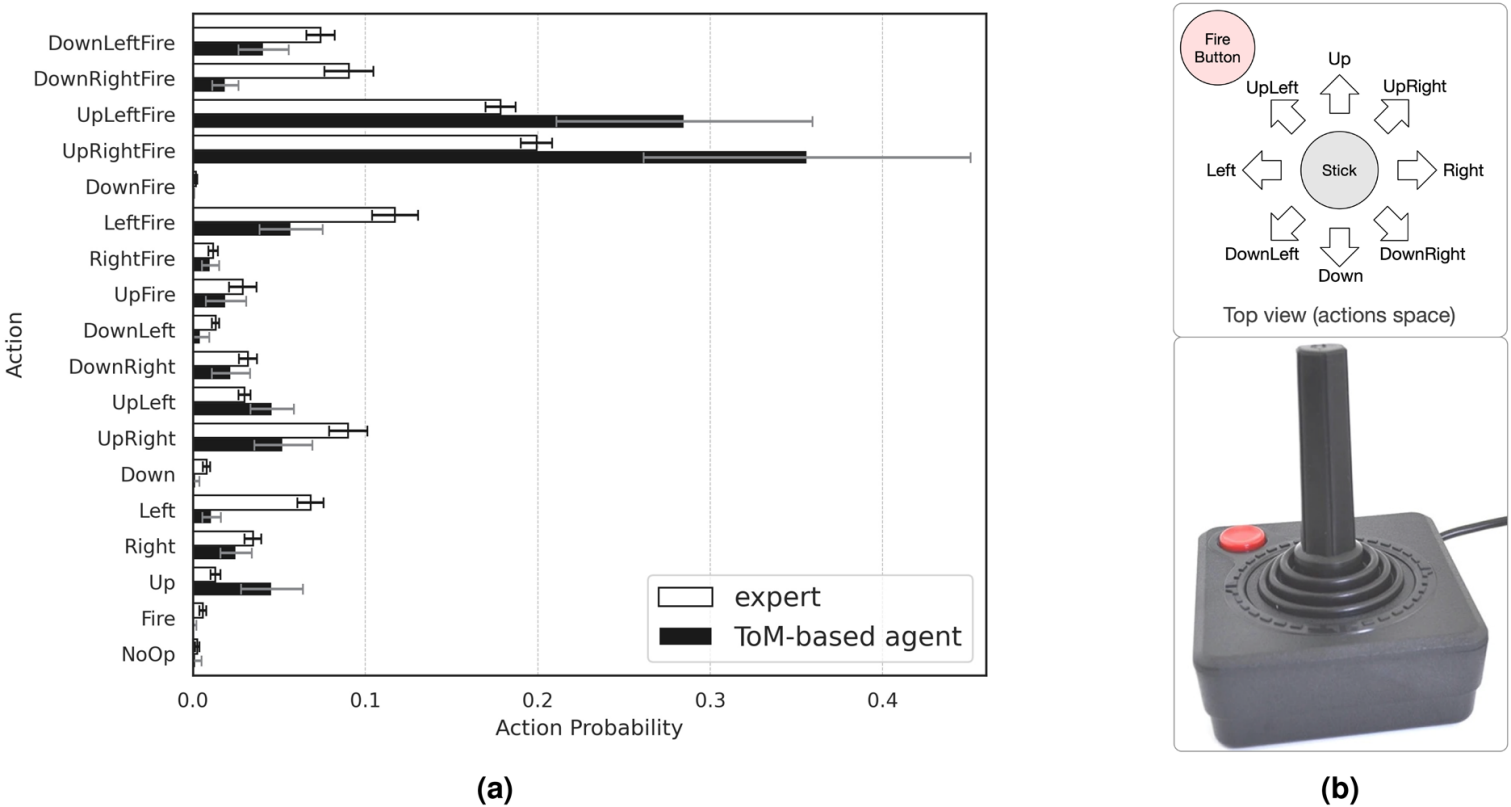
Actions taken by the ToM-based agent and the expert. **(a)** The distribution of the actions chosen by ToM-based agent and expert, averaged over 100 independent runs. Note that the ToM-based agent tries to imitate the expert’s behavior by predicting his goals, rather than copying its actions. **(b)** An Atari 2600 joystick. The joystick has a single button corresponding to fire action, and a stick that can be pushed in one of eight directions. The joystick and the button can be used simultaneously, resulting in a total of 17 possible actions. The player can also neither move nor fire, which is the so-called ‘no action’ in the environment. The image is adapted from amazon.co.uk.

As mentioned before, the lowest bar in the frame, containing information about the score, fuel, and lives, is not encoded and the ToM-based agent is not aware of it, while the expert uses this data to achieve even more scores when possible. The ToM-based agent can not deduce such strategies easily, since it lacks the required information. As expected, the ToM-based agent acts on its false belief of “the expert loses at times with no specific reason.” It tries to avoid similar behaviors that result in those drastic failures, by punishing them extensively. It hereby causes difficulties in learning the whole strategies the expert follows, and contributes to a slight disparity in behavior, as seen in Fig. 3a.

### ToM-based agent vs RL-based agent

To elucidate the aforementioned conclusions and assess the efficacy of the proposed ToM-based learning in SNNs, we trained an agent, with the identical input-output network architecture using R-STDP learning rule, without incorporating mirroring or imitating functionality. We also use the same neural encoding and weight initialization (agent’s prior knowledge) as the ToM-based agent to provide the same information available for both agents. Note that the RL agent should not have access to the expert in this case. This approach allowed the agent to learn how to act in the environment through reinforcement or punishment based on the consequences of its own actions, rather than an external expert. The decision criterion, $\alpha$, is tested in both adaptive and constant manners. Additionally, $\alpha$ was initialized by the value deduced in ToM-based ImRL to facilitate learning under pure RL.

**Figure 4:**
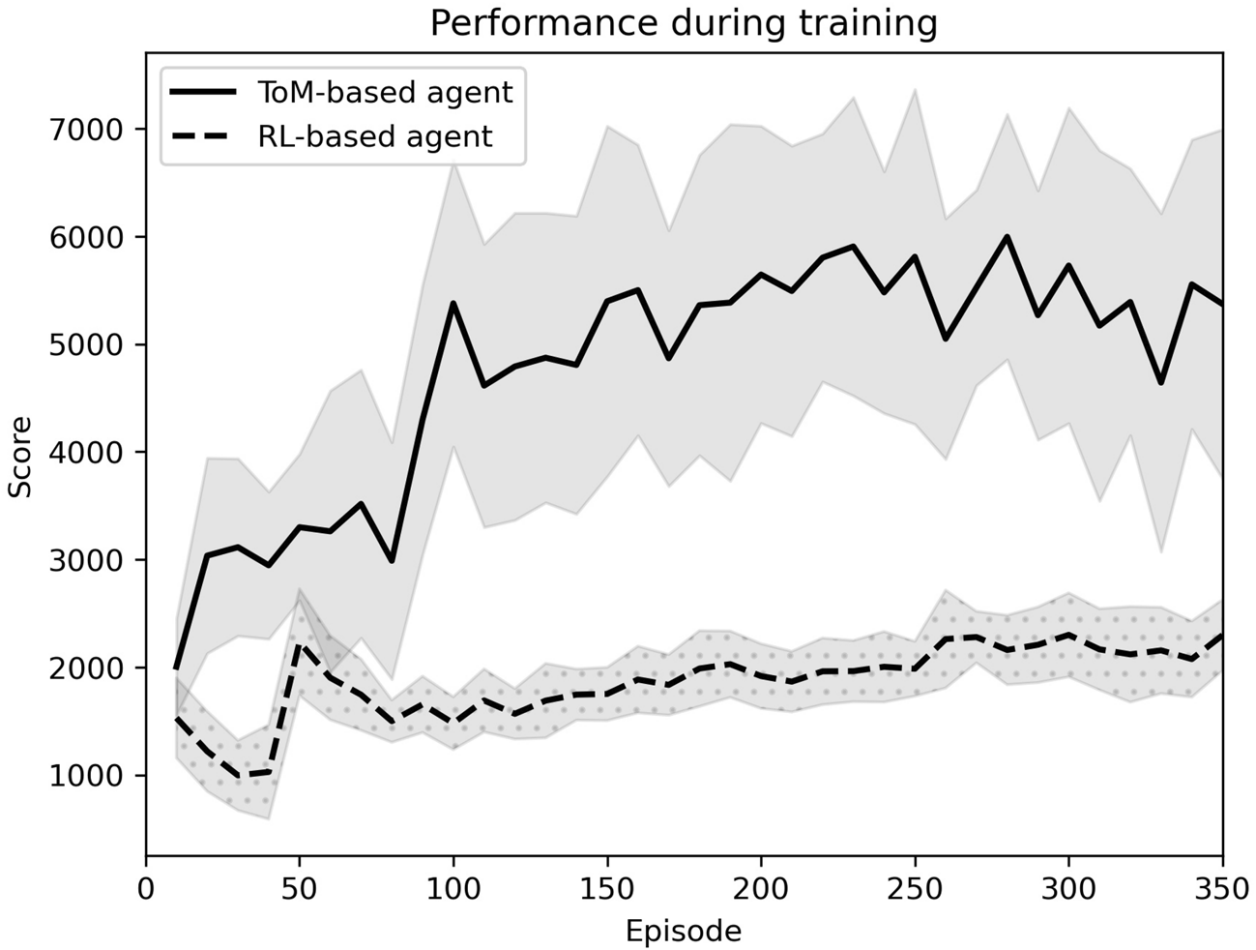
The comparative illustration of ToM-based and RL-based agents throughout the course of learning (significance confirmed by Welch’s T-test: ToM-based agent’s score is larger than the RL-based agent’s score at each episode with $p < 0.001$). Here, the ToM-based agent learns by observing an expert agent achieving an average score of 14,185 in 100 independent runs.

For training the RL-based agent, two reward policies are tested: the case where simple moving along the river is rewarding (same reward setting as in ToM-based ImRL), and the case in which only score achievement is rewarding. As the search space in this environment is relatively large, it takes a huge number of episodes to train the RL-based agent. The results show that none of the above-mentioned reward policies contribute to an agent scoring more than 4000 after training for 800 episodes. Training the RL-based agent using the second reward policy, while employing a constant $\alpha$, demonstrates the best performance among all scenarios. In contrast, utilizing the identical reward policy as the ToM-based agent yields the worst performance, irrespective of the decision criterion. This suggests that reward-engineering poses a lesser challenge in ImRL framework compared to pure RL. Note that the RL-based agent failed to generalize its acquired knowledge sufficiently to emulate a strategy as effective as the expert/human player in River Raid within the same number of episodes as the ToM-based agent. Figure 4 demonstrates the relative performances of the two agents during 350 episodes of training.

## ToM-based ImRL vs. imitation learning

From an AI perspective, ToM-based ImRL framework can be categorized as an imitation learning algorithm, where in general terms, the agent is trained to learn a mapping between observations and actions^37^. This definition embraces a vast number of supervised, unsupervised, and reinforcement learning algorithms. From an RL perspective, imitation learning can be defined as an approach where the reward function is not explicitly defined, but rather are hard-coded in the expert’s demonstrations. IRL^29^, another proposed computational model of ToM, is well interpreted by this definition. However, the definition is not fully aligned with the second assumption of ToM-based ImRL (recall that the ToM-based agent employed its own reward system along with inferring the expert’s intentions).

Essentially, both ToM-based ImRL and IRL are employed in situations where the explicit reward function of the demonstrator is unknown. However, in ToM-based ImRL, the objective is not to make explicit predictions of the expert’s unknown reward function (which is the case in IRL). Instead, it tries to employ its own known reward configuration to infer the expert’s knowledge about the environment, as well as its goals, motivations, and intentions, to complement its own knowledge about the environment. Note that the biological findings supporting the idea of IRL in human brain is still debated. Given the findings of Collette et al. (2017)^38^ which clearly states that IRL is a more promising explanation of social learning rather than traditional imitation learning algorithms in AI, future work can test if the proposed ToM-based ImRL framework can replicate the data better than the best IRL method tested in their study.

There are numerous promising imitation learning algorithms, including behavior cloning, DAgger, generative adversarial imitation learning (GAIL), random expert distillation, disagreement-regularized imitation learning, and many more^39^. Each of these methods has its own advantages and disadvantages which is out of the scope of this paper. What we would like to insist here is that besides their failure in satisfying biological and cognitive findings, they also might be far from practical in real-world scenarios. In a more recent paper by DeepMind, behavior cloning along with self-supervised learning has shown to have an almost human-like performance in a multi-room playhouse environment^40^. However, they have used a massive body of data to train their model which is a big drawback when it comes to real-world problems.

Basically, what ToM-based ImRL framework offers is a reliable approach to imitation learning which is consistent with biological and cognitive findings. No huge amount of data (either synthetic or provided by so many runs of an expert agent) is needed while employing this framework. However, we are still bound to the utility of the expert agent. The expert’s performance is an upper-bound for the ToM-based agent’s performance.

## Discussion

The ToM-based ImRL framework is designed to fulfill the need of effective imitative social learning in interactive environments. In its core, ToM-based ImRL is an input-output SNN, resembling the MNS at its simplest form, employing R-STDP and action mirroring mechanism (via forcing spikes) as a means of associative sequence learning model of imitation. The experimental results in the Atari 2600 River Raid environment shows that this framework enables the learning agent to discover hidden intentions and goals of some expert and learn more effectively than an agent employing pure RL. This framework suggests a bio-inspired imitation learning method, to train an SNN based on ToM and its influence in social learning and cognition.

Classically, there are two main theories about the development of ToM in humans^16^. The first, known as the Theory-Theory, posits that ToM evolves through a cognitive process resembling a theoretical framework, where mental states are deduced from the observable behaviors of individuals^5^. The second theory, the Simulation Theory, asserts that ToM emerges from a cognitive process akin to mental simulation. This theory contends that individuals infer mental states by simulating the cognitive experiences of others within their own minds^3^. Extensive deliberations in the realms of psychology and philosophy have ensued regarding the merit of these divergent theories; however, the Simulation Theory is more compatible with the findings of neuroscience, making it popular among the researchers^3,10^. While conventional computational models of ToM predominantly align with the Theory-Theory, the ToM-based ImRL diverges by aligning more closely with the Simulation Theory; because the ToM-based agent engages in simulating the expert’s behavior internally during the learning phase.

The proposed framework does not account for all capabilities of ToM in humans; but rather focuses on a simplification of the MNS in ToM that influences imitative learning. The framework can be analogous to human ToM only in low-level. Higher-levels of ToM which depends on memory and language require a more complicated network design and training paradigm. However, the model can still be extended to include a neural population that resembles Inferior Parietal Lobule (IPL) by implementing the spatial attention mechanism. This will make the model more similar to the core circuitry of low-level ToM.

The results of the experiments in this study indicate that summarizing the complex circuits of ToM into the simple STS-PMC network under ToM-based ImRL could implement the imitative learning behavior. The ToM-based ImRL framework has enabled the ToM-based agent to identify the goals behind the expert’s actions and to imitate his behaviors rationally. However, the obtained score by the ToM-based agent falls below the achievable score by the expert. There are three main reasons behind this incident. First, the ToM-based agent never observes the result of its inferred action in case of the expert’s failure. So, in case of expert’s failure, the ToM-based agent does not learn what would be the best action, but learns what is the worst to be done in an analogous situation and what might have avoided the failure. Second, the employed encoding scheme in this study has neglected some information about the environment for simplicity in information coding, reducing network complexity, and imposing false belief on the ToM-based agent (not to be mistaken by the false belief task). The expert is trained in a fully-observable environment and its strategies employ all the provided information. Therefore, the ToM-based agent would not be able to hold true beliefs about the expert’s full strategies. The results also depict that the ToM-based agent has used all its capacities to learn the expert’s presumable strategies and behaves accordingly. Third, the proposed framework is too simple to generalize over all possible states in such a complex environment. Adding more neural populations (both in PMC layer and additional hidden layers), along with encoding all the environment’s information and a history of the most recent observations would increase its capacity.

The question we need to answer at this stage is that even if we take care of the above-mentioned issues, would the extended ToM-based ImRL framework obtain a state-of-the-art result in River Raid game. MuZero, the current state-of-the-art algorithm achieving more than 300,000 scores, uses the ordinary tree-based search methods. It does not make any direct constraint on the hidden states to force the agent toward what to learn. Instead, it tries to mirror the structure of a Markov decision process model that computes the expected reward and transition function for a given state and action^41^. ToM-based ImRL at its current simple form cannot achieve such super-human performance. However, using MuZero as the expert should enhance the performance in theory, especially after applying the generalization suggestions. This can be practically tested in future work.

In brief, the proposed ToM-based ImRL emulates the imitation learning inspired by the biological mechanisms in the MNS and reward-motivated learning. It enables the learning agent to infer others’ goals and intentions to further act upon their behavioral strategies. The future direction would be to extend the framework to include more neural populations, representing more complications of the ToM circuits in human brain, and a history of recent events. Given the recent debates on predictive coding and value shaping hypotheses in the context of ToM and social cognition (see^42,43^ for instance), it is also of great importance to test if these hypotheses can be explained by an extended ToM-based ImRL framework.

## Methods

### Spiking neurons

In biological neural populations, the incoming information is processed inside the neuron and then transferred to the other connected neurons through the events over the time, called spikes. These spikes induce an electrical charge/discharge in the post-synaptic neurons. These electrical charges/discharges are integrated over the time and if a threshold is met, a spike is emitted. After each spike event, the membrane potential is reset and the neuron will retain its resting state. Moreover, if the activity in pre-synaptic neurons are not strong enough to excite the post-synaptic neuron toward its spike threshold, its voltage decays back to its resting potential^44,45^.

The behavior of a biological neuron can be roughly modeled by a differential equation and a firing condition. One of the simplest neuronal models is the Leaky Integrate and Fire (LIF), which is formalized as follows:

$$\begin{aligned}&\tau \cdot \frac{du}{dt} = -(u(t)-u_{rest})+R\cdot I(t), \\&\text {if}\, u(t)=\theta \implies \text {Fire}+\text {Reset}, \end{aligned}$$

where *u*(*t*) is the membrane potential of the neuron, $u_{rest}$ is the resting potential, *R* is the membrane resistance, *I* is the input current through time, $\tau$ is the time constant of decaying potential, and $\theta$ is the firing threshold^45^.

### STDP learning rule

For adjusting the synaptic weights between the connected neurons, the well-known Spike-Timing Dependent Plasticity (STDP) learning rule is introduced. STDP is shown to be the neural mechanism behind learning in different regions of the brain. Based on this learning rule, long-term synaptic potentiation/depression depends on the time difference between pre- and post-synaptic spikes. More precisely, STDP states that if the pre-synaptic neuron fires earlier (later) than the post-synaptic one, the synaptic weight is potentiated (depressed)^46^. According to STDP, the weight change could be formalized as follows:

$$\begin{aligned} \Delta w_{ij} = {\left\{ \begin{array}{ll} a_+(w_{ij}).\exp (-|\Delta t_{ij}|/\tau _+) &{}\text {if}\,\Delta t_{ij} \le 0, \\ a_-(w_{ij}).\exp (-|\Delta t_{ij}|/\tau _-) &{}\text {if}\,\Delta t_{ij} > 0, \end{array}\right. } \end{aligned}$$

where $w_{ij}$ is the synaptic weight of the connection from neuron *j* to neuron *i*, $a_\pm$ are the intensity of the synaptic weight changes, $\tau _\pm$ are the time constants defining the learning window, and $\Delta t_{ij} = t_j-t_i$ is the firing time difference between the *i*th and *j*th neurons^45^.

### R-STDP learning rule

To have the reward-motivated learning, the Reward-modulated STDP (R-STDP) learning rule is developed, in which the intensity and polarity of the STDP are influenced by the intensity of reward/punishment signal, established by Dopamine modulation. Since the reward/punishment signal is not immediately received, a decaying eligibility trace is usually used to indicate both the importance of the most recent spikes and the contribution of the earlier spikes in weight changes^46,47^. The R-STDP learning rule is defined by the following differential equations:

4 $$\begin{aligned} \frac{dw}{dt}&= cd, \nonumber \\ \frac{dc}{dt}&= -\frac{c}{\tau _c} + \text {STDP}(\Delta t)\delta (t-t_{pre/post}), \nonumber \\ \frac{dd}{dt}&= -\frac{d}{\tau _d} + \text {DA}(t), \end{aligned}$$

where *c* is the overall dynamics of the effective enzymes (known as synaptic tags) in plasticity, $\tau _{c}$ is the time constant describing effective duration of the synaptic tag, *d* is the dynamic of extracellular Dopamine, DA is the function of Dopamine uptake (also referred to as reward function), and $\tau _d$ is the time constant of Dopamine uptake^45^.

### Spiking neural networks

Spiking neural networks (SNNs) have been the prominent candidate in bio-plausibly modeling of brain mechanisms, such as decision making, object recognition, and action understanding^48–50^. In this variety of artificial neural networks, information flows through binary signals over time. These temporal binary signals are generated by spiking neuron models, like the aforementioned LIF model^51,52^. To feed external sensory input to an SNN, one should interpret the data in terms of voltage, current, or spatio-temporal spikes. In most applications, data is encoded into explicit spike trains and simple non-dynamical input populations are employed to pass the input information to other neural populations in the network. The processed information should also be decoded respectively to convert the neural representations into human-interpretable data^45^.

Neural populations are related to each other according to specified connectivity schemes. Each of these synaptic connections possesses a strength which defines the contribution of the pre-synaptic neuron to the post-synaptic one, and can be either excitatory or inhibitory. The synaptic weights can be adjusted to simulate learning in an SNN. Given the temporal pattern of activity in these networks and to provide a bio-plausible learning, the STDP learning rule and other extensions of it, including the above mentioned R-STDP, are widely employed^46,52^.

### Atari 2600 river raid game

River Raid is considered as a mid-level difficulty game in Atari 2600 series. Back in 1980s, Atari 2600 devices came with a controller containing a stick—which could be moved in 8 directions—and a button. This controller contributed to a total of 18 possible actions in Atari 2600 games, including the River Raid: 8 for moving in any of the 8 directions, 1 for firing, 8 for firing and moving in a direction simultaneously, and 1 for doing nothing.

**Figure 5:**
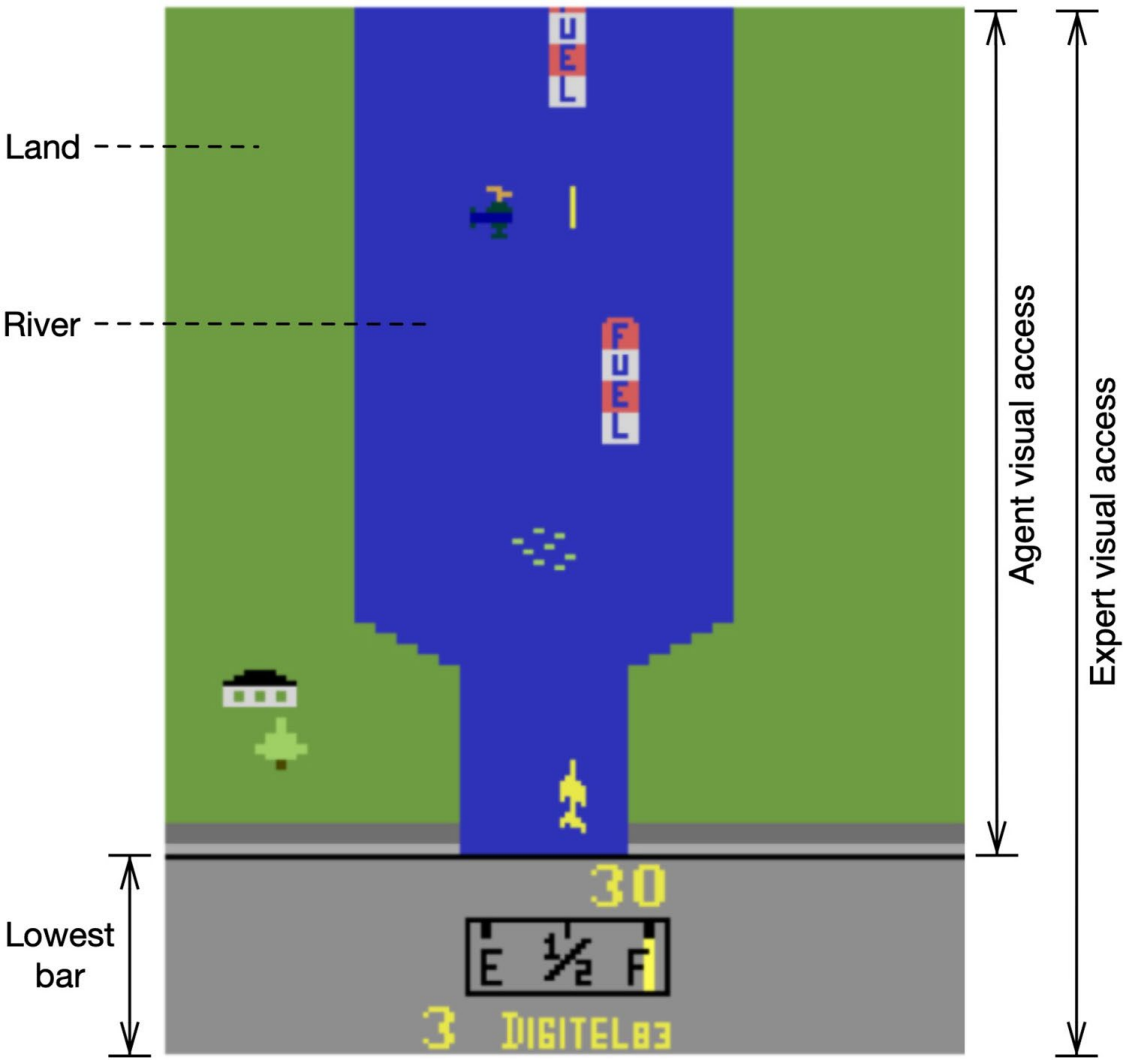
A sample frame of the River Raid game, along with the visual access of ToM-based agent and the expert.

River Raid game takes place in a river, above which a plane, located at the bottom of the frame, is passing. The river passes through green lands on which the plane can not fly (see Fig. 5). The plane faces numerous obstacles and should either avoid them to pass along or shoot them to achieve scores. The obstacles along the river include ships, helicopters, missiles, fuel tanks, and bridges. The missiles are always moving from side to side, whereas the ships and the helicopters are steady at their place or might start moving horizontally on the river. The plane also has a fuel capacity which can be recharged by moving over the fuel tanks. The game is composed of various stages which are depicted by bridges over the river. The player must shoot the bridges to move to the next stage of the game. The higher the stage, the more difficult the game would be. There is also a bar in the lowest part of the frame which includes the number of remaining lives, current score, and the amount of fuel. The player loses the game if the plane collides with an obstacle (excluding the fuel tank) or a green margin, or when it runs out of fuel. In case of failure, an explosion happens, the frame is frozen for moments, and the game resets to the beginning of the last stage.

### Supplementary Information

Supplementary Information.

## Data Availability

The River Raid Atari game environment can be accessed on OpenAI Gym website: https://gymnasium.farama.org/environments/atari/riverraid/. The trained model used as the expert in the experiments can be found on Tensorpack: https://github.com/tensorpack/.
