# Supplementary material for: On computational models of theory of mind and the imitative reinforcement learning in spiking neural networks
Source: Sci Rep. 2024 Jan 23;14:1945. doi: 10.1038/s41598-024-52299-7 (PMC10803361; doi:10.1038/s41598-024-52299-7)
Supplement: Supplementary file 1 — Supplementary Information. [file 41598_2024_52299_MOESM1_ESM.pdf]

On Computational Models of Theory of Mind and the Imitative Reinforcement Learning in Spiking Neural Networks: Supplementary Material

Ashena Gorgan Mohammadi[1] **and Mohammad Ganjtabesh**[1,*]

[1]Department of Computer Science, School of Mathematics, Statistics, and Computer Science, College of Science, University of Tehran, Tehran, Iran
[*]mgtabesh@ut.ac.ir

S1 Cart and Pole Experiment

To justify the ToM-based ImRL framework, a simplified version of it is tested in the Cart and Pole experiment. This experiment is a classic problem in the field of reinforcement learning, where the goal is to balance a pole on a cart by moving the cart to the left or right (Fig. S1). The state of the system is defined by the position of the cart and the angle of the pole. The state is considered failure if the pole falls over too far or if the cart moves too far from the center of the track.

The ToM-based agent in this experiment employs the input-output SNN with four input neurons and two output neurons. The output neurons represent the left and right actions. The first two input neurons represent the angle of the pole and the other two correspond to its angular velocity. The encoding of the input values in each of these pairs of neurons is as follows. Let x be a floating-point value representing the input (angle/angular velocity), and let T be the duration of input presentation. First, the input values are discretized into T bins; so x will be mapped to a value, say x', in the range $[0, T-1]$. Then, the first neuron will spike at time x' and the second neuron (or the complement neuron) will spike at time $T-x'$. Note that this encoding scheme also employs both temporal and spatial information.

The agent is trained to balance the pole on the cart by observing an expert trained by Hill-climbing algorithm. For the mirror mechanism, the output neuron corresponding to the expert's action fires forcefully at time $\lfloor T/2 \rfloor$. Together with the above-mentioned encoding scheme, the mirror mechanism lets the training to be done in a single learning phase, since the agent can simultaneously learn to inhibit self-action via R-STDP. The ToM-based agent receives a reward of +1 for each time step that the pole remains upright and receives a reward of -1 when the pole falls over.

The agent is trained in two cases: 1) the expert performs random actions with a probability that is reduced through time, and 2) the expert always plays perfectly. As Fig. S2 shows, the ToM-based agent masters the task by observing the first expert within 25 episodes of training and by observing the perfect expert for only one episode; whereas an agent trained by the standard RL algorithm (Q-learning) requires more than 100 episodes to learn the task. This result shows that the ToM-based agent can learn the task more efficiently by observing the expert and inferring its goals and actions. The code is available at `https://github.com/atenagm1375/cartpole`.

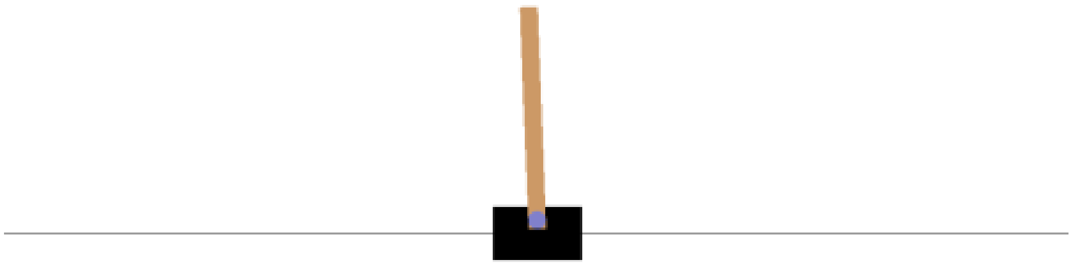

Fig. S1. The Cart and Pole environment.

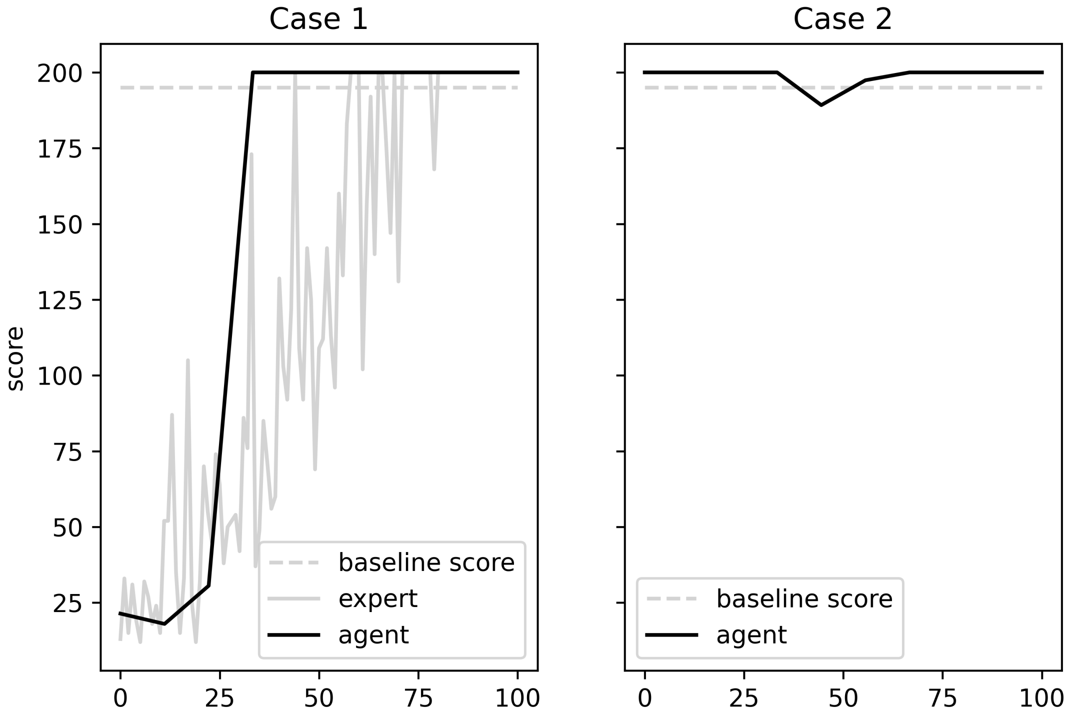

Fig. S2. Score of the ToM-based agent after each training episode while observing an expert in two cases: Case 1 corresponds to an expert performing random actions at the beginning, and Case 2 indicates an expert performing perfect actions (no random actions).
